# pH-responsive hydrogel facilitates skin penetration of extracellular vesicle-like nanovesicles for enhanced atopic dermatitis therapy

**DOI:** 10.1093/rb/rbag196

**Published:** 2026-09-03

**Authors:** Kaizheng Li, Mengnan Dai, Chao Xu, Zengjie Fan

**Affiliations:** Key Laboratory of Dental Maxillofacial Reconstruction and Biological Intelligence Manufacturing of Gansu Province, School of Stomatology, Lanzhou University, Lanzhou 730000, China; Key Laboratory of Dental Maxillofacial Reconstruction and Biological Intelligence Manufacturing of Gansu Province, School of Stomatology, Lanzhou University, Lanzhou 730000, China; Department of Oral Implantology, Gansu Provincial People’s Hospital, Lanzhou 730000, China; Key Laboratory of Dental Maxillofacial Reconstruction and Biological Intelligence Manufacturing of Gansu Province, School of Stomatology, Lanzhou University, Lanzhou 730000, China

**Keywords:** atopic dermatitis, pH-responsive hydrogel, plant-derived extracellular vesicle-like nanovesicles, transdermal delivery

## Abstract

Atopic dermatitis (AD) is a chronic inflammatory skin disorder, and the long-term use of topical corticosteroids is often limited by adverse effects and poor tolerability. Herein, we developed CAAM-G, a corticosteroid-free hydrogel platform composed of carboxymethyl cellulose, acrylamide and acrylic acid, and loaded with *Gentiana macrophylla* Pall.-derived extracellular vesicle-like nanovesicles (GELNs) and L-menthol. The hydrogel exhibited favorable mechanical properties, tissue adhesion and swelling capacity. Under mildly to moderately alkaline conditions relevant to lesional AD skin, it exhibited pH-dependent release of GELNs and menthol. The released menthol enhanced the cutaneous distribution of PKH26-labeled GELN-associated fluorescence in a porcine skin Franz diffusion model. *In vitro*, GELN-containing formulations efficiently scavenged reactive oxygen species, suppressed pro-inflammatory cytokine secretion and promoted macrophage polarization toward an M2-like phenotype. In the 2,4-dinitro-1-fluorobenzene-induced AD-like mouse model, the high-dose formulation, CAAM-GH, significantly alleviated ear swelling, epidermal thickening, mast cell infiltration and other AD-like manifestations. For several evaluated endpoints, CAAM-GH produced greater improvement than the dexamethasone reference group. No evident treatment-associated systemic toxicity was detected under the experimental conditions. Integrated transcriptomic and Western blot analyses indicated suppression of nuclear factor-kappa B (NF-κB)-associated pro-inflammatory signaling. These findings support CAAM-G as a promising corticosteroid-free topical platform for the treatment of AD, although further pharmacological and toxicological evaluation is required before clinical translation.

## Graphical Abstract

**Graphical Abstract:**
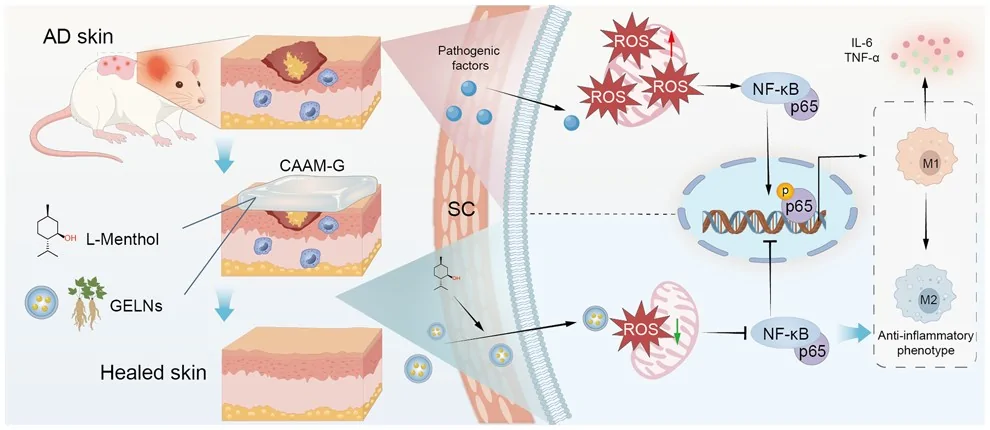

## Introduction

Atopic dermatitis (AD) is one of the most prevalent chronic inflammatory skin diseases worldwide, characterized by intense pruritus, recurrent eczematous lesions, epidermal barrier dysfunction and immune dysregulation [1, 2]. The crosstalk between skin barrier disruption and immune imbalance constitutes the central pathological axis of AD, in which the balance between pro-inflammatory M1 and anti-inflammatory M2 macrophage polarization plays a critical regulatory role [3–5]. Excessive accumulation of reactive oxygen species (ROS) in lesional skin further amplifies M1 polarization and pro-inflammatory cytokine secretion, forming a self-perpetuating pathological cycle that sustains chronic inflammation and impairs barrier repair [6]. Simultaneously restoring macrophage M1/M2 balance and eliminating oxidative stress therefore represents a mechanistically grounded therapeutic strategy for AD [7, 8].

Current first-line treatments, including topical corticosteroids and calcineurin inhibitors, are limited by significant adverse effects upon long-term use, while emerging biologics targeting the interleukin-4 (IL-4) receptor axis remain restricted by high cost and parenteral administration [2, 9]. These limitations underscore an urgent need for safe, cost-effective and mechanistically targeted topical formulations. Plant-derived extracellular vesicle-like nanovesicles (PENs) have recently emerged as promising therapeutic candidates for inflammatory skin diseases. As naturally derived lipid bilayer vesicles enriched with bioactive cargo, including miRNAs, functional proteins and flavonoids, PENs possess intrinsic anti-inflammatory and immunomodulatory activities, together with excellent biocompatibility and negligible immunogenicity [10–12]. Recent studies have demonstrated their therapeutic potential in AD, with *Portulaca oleracea*-derived nanovesicles reported to restore M1/M2 polarization balance through inhibition of the nuclear factor kappa-B (NF-κB) and stimulator of interferon genes (STING) pathways [13]. These findings suggest that PENs derived from medicinal plants with well-established anti-inflammatory properties may represent a valuable but underexplored source of topical nanotherapeutics for AD. *Gentiana macrophylla* Pall. (Qinjiao), a traditional Chinese medicinal herb, has long been used for inflammatory disorders, and its major bioactive constituent gentiopicroside has been shown to suppress NF-κB signaling by blocking inhibitor of nuclear factor kappa-B kinase subunit beta (IKKβ) phosphorylation [14–16]. Based on this pharmacological background, *G.macrophylla* represents a rational botanical source for PEN development. However, whether *G.macrophylla*-derived extracellular vesicle-like nanovesicles (GELNs) retain anti-inflammatory bioactivities and can modulate macrophage polarization in AD remains unknown—a gap this study directly addresses.

Despite their therapeutic promise, the topical application of PENs remains challenged by limited retention at lesion sites, the formidable barrier function of the stratum corneum, and insufficient penetration into inflamed skin [17]. These barriers are particularly relevant for AD therapy, where effective modulation of local oxidative stress, inflammatory signaling and macrophage polarization requires sustained delivery of bioactive vesicles to the pathological microenvironment. Hydrogels provide a suitable topical delivery matrix because of their high water content, tissue adhesiveness, lesion conformability and capacity for localized and sustained cargo release [18]. Furthermore, stimuli-responsive hydrogels can be designed to release therapeutic agents in response to disease-associated microenvironmental cues, thereby improving the spatial precision of topical therapy. Moreover, incorporating chemical penetration enhancers into these hydrogel matrices represents a common strategy to temporarily overcome the stratum corneum, thereby driving the transdermal flux of nano-cargoes. To address these challenges, we developed GELNs-loaded hydrogels (CAAM-G), a hydrogel platform that delivers therapeutic agents via a coordinated dual-action mode: on-demand payload release combined with localized enhancement of drug penetration. The polymer network enables preferential release of GELNs and L-menthol under mildly alkaline conditions, consistent with the reported microenvironment of AD lesions [19, 20]. Within this platform, L-menthol acts as a stratum corneum modulator that transiently disrupts ordered lipid packing, thereby facilitating the cutaneous distribution of the loaded therapeutic components [21]. Ultimately, this study establishes a potentially generalizable topical delivery strategy. By integrating a pH-responsive delivery matrix, a penetration-enhancing component and repair-promoting plant-derived nanovesicles, this approach may improve therapeutic efficacy in AD and provide a basis for the development of topical treatments for inflammatory skin disorders.

## Materials and methods

### Botanical materials and chemical reagents

Fresh roots of *G.macrophylla* Pall. were purchased from a local market in Lanzhou, China. Carboxymethyl cellulose (CMC), acrylamide (AAm), acrylic acid (AA), N,Nʹ-methylenebisacrylamide (MBA), ammonium persulfate (APS) and L-menthol were purchased from Sigma-Aldrich (St. Louis, MO, USA). Lipopolysaccharide (LPS), phosphate-buffered saline (PBS), 2,2-diphenyl-1-picrylhydrazyl (DPPH), 2,2′-azino-bis(3-ethylbenzothiazoline-6-sulfonic acid) (ABTS) assay kit, 2′,7′-dichlorodihydrofluorescein diacetate (DCFH-DA) probe, dihydroethidium (DHE) probe, bicinchoninic acid (BCA) protein assay kit, cell counting kit-8 (CCK-8), 5-ethynyl-2′-deoxyuridine (EdU) cell proliferation kit, terminal deoxynucleotidyl transferase-mediated dUTP nick-end labeling (TUNEL) apoptosis detection kit, enzyme-linked immunosorbent assay (ELISA) kits for interleukin-6 (IL-6), tumor necrosis factor-α (TNF-α) and immunoglobulin E (IgE), PKH26 fluorescent dye, alanine aminotransferase (ALT) assay kit, aspartate aminotransferase (AST) assay kit, blood urea nitrogen (BUN) assay kit, creatinine (CRE) assay kit, primary antibodies against filaggrin, loricrin, involucrin, β-actin, p65, p-p65 and horseradish peroxidase (HRP)-conjugated secondary antibodies were obtained from Solarbio (Beijing, China). 2,4-Dinitro-1-fluorobenzene (DNFB) and dexamethasone acetate were obtained from Macklin (Shanghai, China). All other reagents were of analytical grade.

### Preparation of GELNs

GELNs were isolated from fresh *G.macrophylla* Pall. roots by differential centrifugation as previously described [10]. Briefly, the roots were mechanically homogenized in ice-cold PBS and filtered through multilayer gauze (Figure 1A). The filtrate was subjected to three successive rounds of centrifugation at 10 000 × g for 60 min each at 4°C to remove debris and large vesicles. The resulting supernatant was collected, and the purified GELNs were stored at −80°C until use.

**Figure 1:**
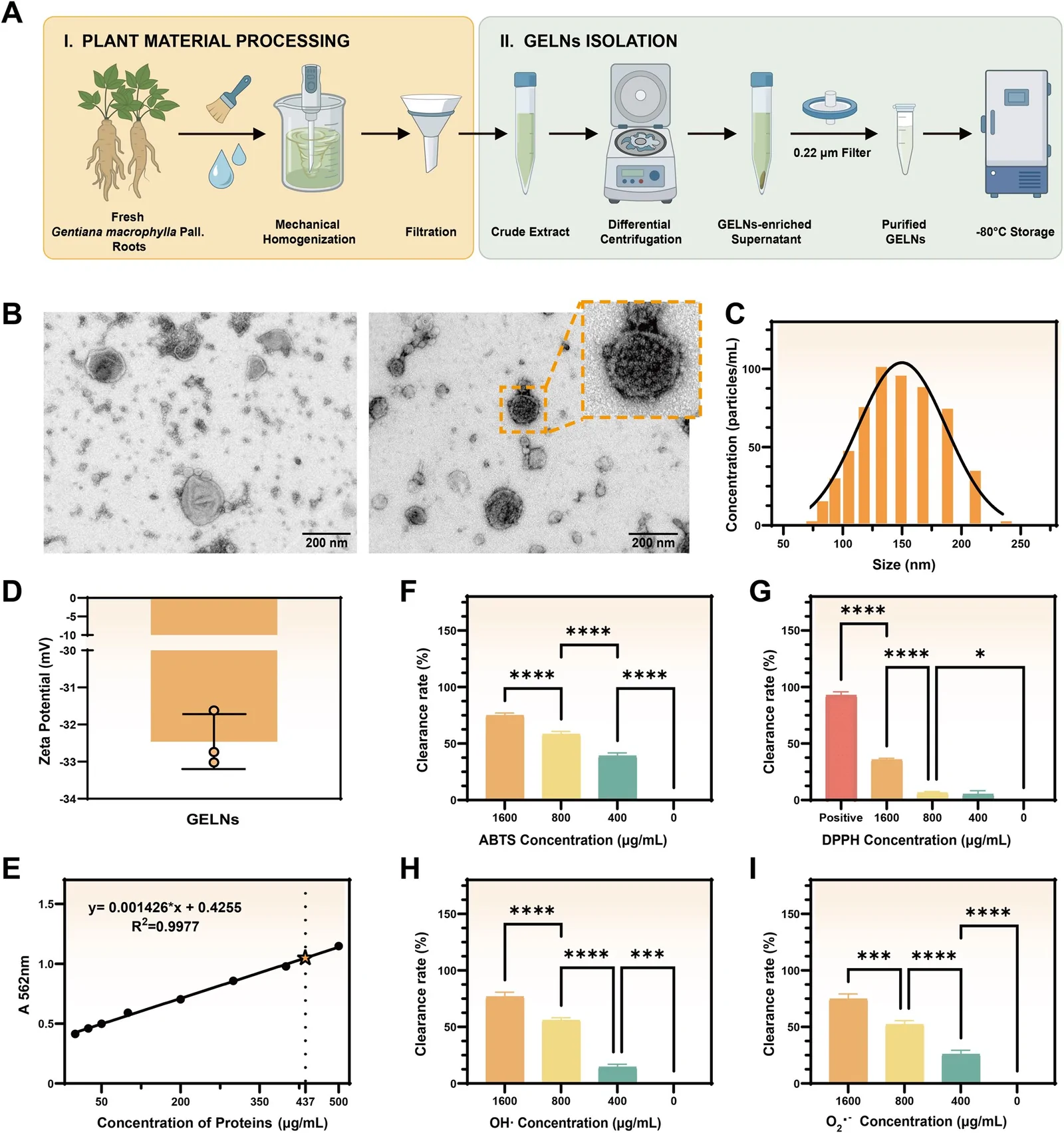
Preparation and characterization of GELNs. (**A**) Schematic flowchart of GELN isolation and purification. (**B**) Representative TEM images showing intact lipid bilayer morphology. Scale bar: 200 nm. (**C**) Particle size distribution by DLS. (**D**) Zeta potential. (**E**) BCA protein standard curve and quantification of protein concentration. Radical scavenging rates against ABTS (**F**), DPPH (**G**), ·OH (**H**), and ·O_2_^−^ (**I**) at 0, 400, 800 and 1600 μg/mL. Data are presented as mean ± SD; ns, not significant; *p < 0.05, **p < 0.01, ***p < 0.001, ****p < 0.0001.

### Preparation of CAAM-G hydrogels

AA and L-menthol were mixed at defined molar ratios and reacted at 70°C for 5 min. The resulting AA–menthol mixture was subsequently used for hydrogel synthesis. This thermal treatment facilitated esterification between the carboxyl group of AA and the hydroxyl group of menthol, enabling the covalent integration of menthol into the polymer network and ensuring controlled release kinetics [22]. CMC (2 g) was dissolved in deionized water (40 mL) under magnetic stirring for 12 h, followed by the addition of AAm, the AA–menthol solution, MBA (0.1 g) and APS (0.2 g) [23]. Polymerization was carried out at 40°C under continuous stirring. The resulting hydrogels were dialyzed against distilled water (6 L, refreshed twice daily) for 72 h to remove unreacted monomers and the crosslinker and were then lyophilized. Hydrogel formulations with varying CMC/AA/AAm mass ratios are summarized in Table 1. CAAM-G were prepared by immersing lyophilized CAAM233 in PBS containing GELNs at 800 μg/mL (CAAM-GL) or 1600 μg/mL (CAAM-GH) until swelling equilibrium was reached at 4°C; these concentrations were selected based on the cytocompatibility results showing maintained cell viability above 80% up to 1600 μg/mL (Section Cytocompatibility, proliferation and migration). A GELNs-loaded hydrogel without menthol (CAA-GH) was prepared identically using the CAAM233 formulation without the AA–menthol pre-reaction step, serving as a control to isolate the contribution of menthol-mediated transdermal enhancement.

**Table 1:** The CAAM hydrogels with different formulations.

Hydrogel number	CMC/AA/AAm mass ratio
CAAM222	2:2:2
CAAM223	2:2:3
CAAM232	2:3:2
CAAM224	2:2:4
CAAM242	2:4:2
CAAM233	2:3:3
CAAM234	2:3:4
CAAM243	2:4:3
CAAM244	2:4:4

### Characterization of GELNs and CAAM hydrogels

The morphology of GELNs was examined by transmission electron microscopy (TEM; Tecnai G2 Spirit Bio-TWIN, FEI, USA) after negative staining with 2% phosphotungstic acid [13]. Their hydrodynamic diameter and zeta potential were measured by dynamic light scattering (DLS NanoBrook 90Plus PALS, Brookhaven Instruments, USA). Total protein content was quantified by the BCA assay. The chemical structures of CAAM hydrogels were confirmed by fourier-transform infrared (FTIR) spectroscopy (Nicolet iS50, Thermo Fisher Scientific; 4000–400 cm^−1^). Hydrogel pore morphology was examined by SEM (lack manufactuer and country) after lyophilization and gold sputter-coating.

### Antioxidant activity of GELNs

ROS scavenging activity was evaluated using ABTS, DPPH, hydroxyl radical (·OH) and superoxide anion (·O_2_^−^) assays with GELN concentrations of 0–1600 μg/mL. For ABTS, absorbance was measured at 734 nm; for DPPH, absorbance was measured at 517 nm after a 30 min incubation; for ·OH, absorbance was measured via TMB oxidation at 645 nm; and for ·O_2_^−^, absorbance was measured via pyrogallol autoxidation at 325 nm. Scavenging rates were calculated as:


(1) Scavenging rate (%)=[(A_control−A_sample)/A_control]×100%

### Mechanical properties and swelling behavior

Tensile and compressive properties were measured using a universal testing machine (CMT2102, Shandong Wanchen, China) at speeds of 10 and 5 mm/min, respectively, on dumbbell-shaped specimens (working length 25 mm, width 4 mm, thickness 2 mm). Swelling ratios were determined by immersing hydrogel samples (5 mm × 5 mm × 2 mm) in PBS at pH 5.5, 7.5 and 8.5 at 37°C for 24 h, and were calculated as:


(2) SR (%)=(W_s−W_d)/W_d×100%,

where *W*_s and *W*_d represent the swollen and dry weights, respectively.

### *In vitro* drug release

CAAM-G hydrogels were immersed in PBS (30 mL; pH 5.5, 6.0, 6.5, 7.0, 7.5, 8.0 or 8.5) at 37°C with shaking at 150 rpm. At designated time points, 5-mL aliquots were withdrawn and replaced with fresh PBS. Released GELNs were quantified by the BCA assay, and menthol release was determined spectrophotometrically.


(3) Hemolysis rate (%)=[(A_sample−A_negative)/(A_positive−A_negative)]×100%

### Cell culture

HaCaT keratinocytes and RAW 264.7 macrophages were obtained from the National Collection of Authenticated Cell Cultures (Shanghai, China) and maintained in DMEM supplemented with 10% FBS and 1% penicillin–streptomycin at 37°C in 5% CO_2_.

### Cytocompatibility, proliferation and migration

Cell viability was assessed by CCK-8 assay. HaCaT cells were treated with GELNs (0–1600 μg/mL) for 1, 3, 5 and 7 days, or with menthol-loaded hydrogels (0–50% menthol) for 24 h. Proliferative activity was evaluated by the EdU incorporation assay. Cell migration was assessed by scratch assay: confluent HaCaT monolayers were scratched, washed and cultured in serum-free medium containing test formulations; wound closure was recorded at 0, 6 and 12 h.

### Cellular uptake

GELNs were labeled with PKH26 (10 μM) and recovered by ultracentrifugation (150 000 × g, 1.5 h). The labeled vesicles were washed to remove residual unbound dye before use. HaCaT cells were incubated with PKH26-GELNs or hydrogel formulations for 6 h, fixed with 4% paraformaldehyde and counterstained with DAPI for fluorescence microscopy.

### *In vitro* transdermal penetration

Transdermal permeability was evaluated using a modified Franz diffusion cell system (RYJ-12B, Shanghai Huanghai, China; effective area 2.2 cm²) with porcine skin as the membrane. Using PKH26-GELNs, CAAM-PKH26-GELNs hydrogels were prepared containing 0%, 1%, 5% and 10% menthol. The pure PKH26 and CAAM-PKH26-GELNs were applied to the skin surface, and the receptor compartment contained PBS at 37°C. At 1, 3, 6 and 12 h, the skin samples were collected, cryofixed, sectioned and imaged by fluorescence microscopy. Maximum penetration depth and overall fluorescence distribution were analyzed using ImageJ.

### Intracellular ROS detection

Intracellular ROS levels were assessed by DCFH-DA fluorescence staining. HaCaT cells were treated with test formulations, incubated with 10 μM DCFH-DA for 30 min at 37°C, and fluorescence intensity was quantified using ImageJ.

### Anti-inflammatory effects and macrophage polarization

RAW 264.7 cells were stimulated with LPS to establish an inflammatory model. After 24 h of treatment with test formulations, IL-6 and TNF-α concentrations in cell supernatants were quantified by ELISA. For polarization analysis, cells were stained with FITC-conjugated anti-CD86 and PE-conjugated anti-CD206 antibodies at 4°C for 30 min and analyzed by flow cytometry (FACSCanto, BD Biosciences, USA).

### AD mouse model and *in vivo* efficacy

All animal procedures were approved by the Ethics Committee of the School of Stomatology, Lanzhou University (LZUKQ-2026-026) and conducted in accordance with the National Institutes of Health Guide for the Care and Use of Laboratory Animals. Female BALB/c mice (18–20 g, SPF grade) were housed under standard conditions (12 h light/dark cycle, 22 ± 2°C, 50 ± 10% relative humidity) with *ad libitum* access to standard rodent chow and sterilized water, and acclimatized for 1 week prior to experimentation. Mice were randomly assigned to seven groups (*n* = 5 per group). The sample size was determined based on prior studies using the DNFB-induced AD model, which have consistently demonstrated sufficient statistical power to detect treatment effects at this group size [24, 25]. AD was induced by topical application of 1% DNFB in acetone/olive oil (4:1 v/v) on shaved dorsal skin (Day 1), followed by 0.5% DNFB challenges every 3 days (Days 5–14) (Figure 5A) [26]. Test formulations or dexamethasone (10 mg/kg) were administered topically eight times over the treatment period. Dermatitis scores (0–3 for each of erythema, hemorrhage, edema and scaling; maximum total score 12) were assessed by two independent observers blinded to group assignment throughout the experimental period. For the ear edema model, the right ears were challenged with DNFB and treated for 4 days; ear thickness and erythema scores were recorded daily [27, 28].

### Histological, immunohistochemical and immunofluorescence analysis

Paraffin-embedded skin sections (4 μm) were stained with hematoxylin and eosin (H&E) and toluidine blue to evaluate epidermal thickness and mast cell infiltration, respectively. For immunohistochemistry (IHC), sections were incubated with primary antibodies against CD86 and CD206 overnight, followed by DAB development and hematoxylin counterstaining. For IF, sections were stained with DHE to detect ROS, TUNEL to detect apoptosis, or antibodies against occludin-1 and claudin-1, followed by incubation with Alexa Fluor 594-conjugated secondary antibodies and DAPI counterstaining.

### Serum IgE detection, serum biochemical measurement and hemolysis assays

The blood was centrifuged at 3000 × g for 15 min to separate the serum and was stored at −80°C for biochemical analyses. ELISA kit were used to measure serum IgE levels to evaluate systemic allergic inflammatory status. Four biochemical assay kits were utilized to detect serum ALT, AST, BUN and CRE. Erythrocytes isolated from BALB/c mice were prepared as a 2% suspension in physiological saline. Samples were incubated with test formulations at 37°C for 3 h, and the hemolysis rate was determined spectrophotometrically at 540 nm, with 0.1% Triton X-100 as the positive control.

### Transcriptomic analysis and Western blot

For transcriptomics, total RNA was extracted from skin tissue using TRIzol, quantified using a NanoDrop spectrophotometer and sequenced on the Illumina NovaSeq 6000 platform. Differentially expressed genes (DEGs) were identified using thresholds of |log_2_FoldChange| > 1 and *P *< 0.05, followed by GO, KEGG and GSEA enrichment analyses. For Western blot, total protein was extracted in RIPA buffer, separated by SDS-PAGE, transferred to PVDF membranes and probed with primary antibodies against filaggrin, loricrin, involucrin, p-p65, p65 and β-actin (1:1000 dilution), followed by HRP-conjugated secondary antibodies (1:5000). Bands were visualized by ECL and quantified relative to β-actin using ImageJ.

### Statistical analysis

All data are presented as mean ± SD (*n* ≥ 3). Statistical comparisons were performed using one-way ANOVA with Tukey’s *post hoc* test in GraphPad Prism 9.0. Significance thresholds were defined as follows: *P < 0.05, **P < 0.01, ***P < 0.001, ****P < 0.0001; ns, not significant.

## Results

### Characterization and antioxidant capacity of GELNs

The isolated and purified GELNs (initial concentration: 1600 μg/mL) were systematically characterized. TEM imaging revealed that GELNs displayed typical extracellular vesicle-like morphology with intact lipid bilayer structures, presenting as uniform spherical or ellipsoidal vesicles (Figure 1B). DLS analysis showed a monodisperse size distribution with a peak diameter of 132.62 nm (Figure 1C), and zeta potential measurement indicated a surface charge of −32.46 ± 0.74 mV (Figure 1D), consistent with colloidal stability criteria for plant-derived nanovesicles [10, 11]. BCA protein quantification determined a protein concentration of 436.68 μg/mL in freshly isolated GELNs (Figure 1E), reflecting a protein-rich vesicular cargo consistent with previously reported PENs [10, 11].

GELNs demonstrated concentration-dependent radical scavenging activity across ABTS, DPPH, ·OH and ·O_2_^−^ assays over a concentration range of 0–1600 μg/mL (Figure 1F–I), with statistically significant differences among the concentration groups (*P *< 0.0001). These results establish GELNs as intrinsically bioactive particles with antioxidant capacity relevant to the ROS-rich environment of AD lesional skin.

### Characterization of CAAM hydrogel

FTIR analysis confirmed successful hydrogel network formation and menthol incorporation. A new characteristic peak at 1160 cm^−1^, assigned to C–O stretching of the tertiary alcohol in menthol, appeared in the CAAM spectrum but was absent in CAA, while all other characteristic bands of CMC, acrylic acid and acrylamide were retained (Figure 2A) [29]. SEM imaging revealed a well-defined interconnected 3D porous network across all formulations, with pore diameter decreasing progressively as AA and AAm concentrations increased from CAAM222 to CAAM244 (Figure 2B and C). This reduction in pore size was likely attributable to the increased polymer content, enhanced chain entanglement, and stronger intermolecular interactions, which produced a denser network with a smaller effective mesh size.

**Figure 2:**
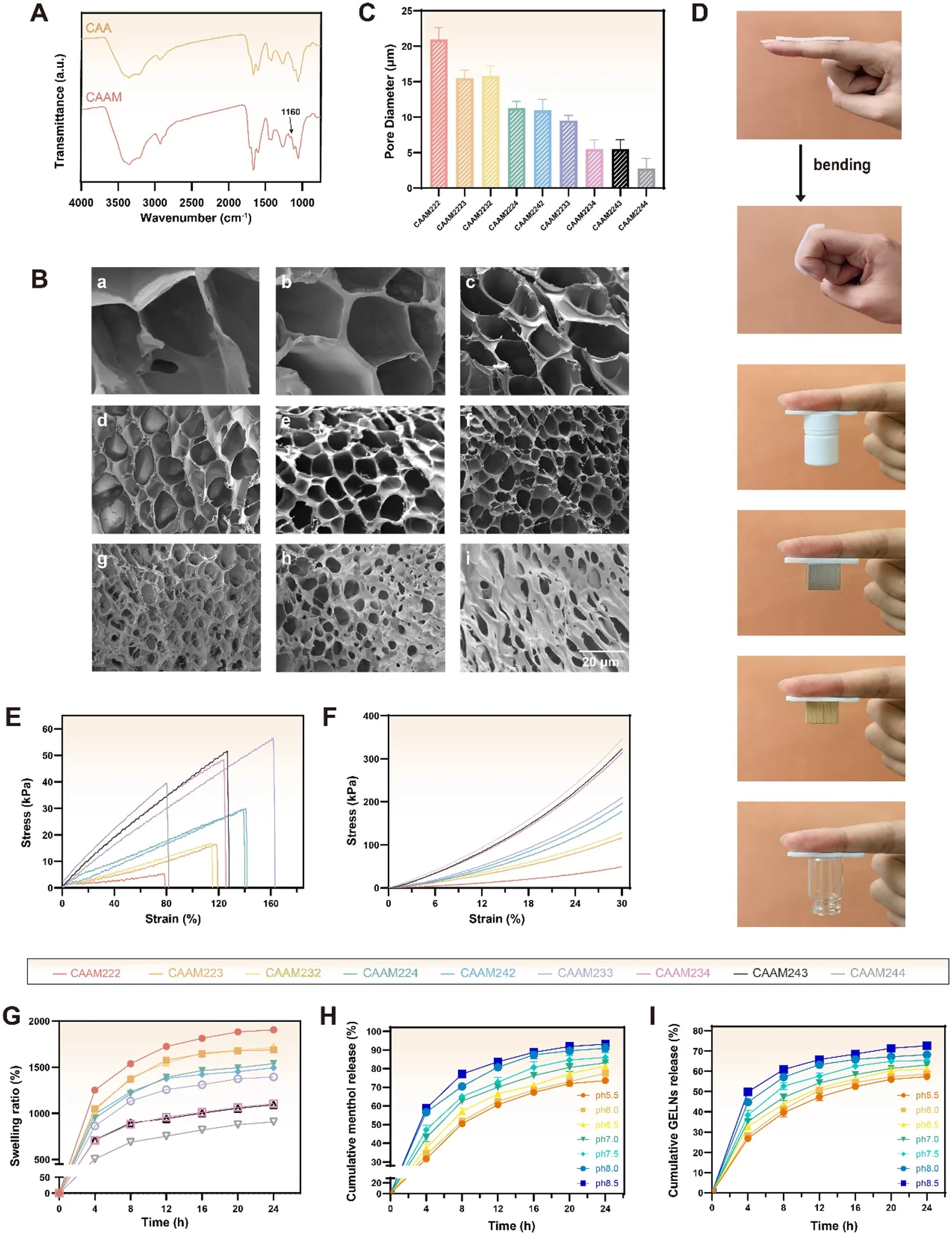
Characterization and properties of CAAM hydrogels. (**A**) FTIR spectra of CAA and CAAM hydrogels. (**B**) SEM images of CAAM hydrogel formulations: (**a**) CAAM222, (**b**) CAAM223, (**c**) CAAM232, (**d**) CAAM224, (**e**) CAAM242, (**f**) CAAM233, (**g**) CAAM234, (**h**) CAAM243, (**i**) CAAM244. Scale bar: 20 μm. (**C**) Pore diameter across formulations. (**D**) Photographs demonstrating skin adhesion and deformation adaptability of CAAM233 on a human fingertip and adhesion to glass, plastic, wood and metal substrates. (**E**) Tensile and (**F**) compressive stress–strain curves. (**G**) Swelling ratio over 24 h. (**H**) Cumulative menthol release and (**I**) GELNs release at pH 5.5, 6.0, 6.5, 7.0, 7.5, 8.0 and 8.5 over 24 h.

### Mechanical properties of CAAM hydrogel

The hydrogel conformed precisely to the skin curvature during dynamic fingertip bending without debonding, exhibited robust adhesion to glass, plastic, wood and metal substrates (Figure 2D), supporting its suitability for use as a topical skin dressing. In the tensile tests, the ultimate tensile strains of the formulations were between 70% and 170%, while the peak stress values ranged from 5 to 60 kPa. The CAAM233, CAAM224 and CAAM242 formulations exhibited significantly higher tensile strains, but only CAAM233 exhibited a peak stress that exceeded 55 kPa. In the compression tests, none of the hydrogel samples exhibited yielding or fracture within the strain range of 0–30%, indicating excellent compressive resilience and structural integrity. The initial slope of the stress–strain curve increased significantly with increasing cross-linking density, demonstrating enhanced stiffness and resistance to elastic deformation. At 30% strain, the compressive stress of the CAAM244 sample reached nearly 350 kPa, whereas that of CAAM222 was only ∼50 kPa, indicating significant differences in compressive load-bearing capacity across the formulations. Among all formulations, CAAM233 demonstrated the best overall mechanical performance, achieving peak tensile stress exceeding 55 kPa while retaining high extensibility (Figure 2E), and maintaining structural integrity under 30% compressive strain (Figure 2F).

### Swelling behavior and *in vitro* drug release

All CAAM formulations exhibited a consistent three-phase swelling profile, comprising rapid swelling (0–4 h), slower swelling (4–12 h) and equilibrium swelling (≥12 h), with equilibrium swelling ratios exceeding 900% for all formulations (Figure 2G). CAAM233 exhibited a favorable balance of swelling capacity and mechanical performance and was selected for subsequent experiments.

Both menthol and GELNs showed pH-dependent release, with higher cumulative release observed at higher pH values (Figure 2H and I). At 24 h, cumulative menthol release reached ∼95% at pH 8.5 and 75% at pH 5.5, whereas the cumulative release of GELNs exceeded 80% at pH 8.5 and ∼60% at pH 5.5. This graduated pH-dependent release profile suggests that the encapsulated components may be released more readily in the relatively alkaline microenvironment associated with lesional AD skin.

### Cytocompatibility and proliferative activity

GELNs maintained cell viability above 80% at lower concentrations and promoted progressive proliferation at higher concentrations (1400–1600 μg/mL), reaching over 140% by Day 7 (Figure 3A). Based on the favorable proliferative activity observed at the upper end of this concentration range, 1600 μg/mL GELNs was selected as the high-dose concentration for hydrogel preparation. Menthol-loaded hydrogels containing 0%, 5% and 10% menthol maintained cell viability above 80%, whereas viability declined to ∼70% and 50% at 20% and 50% menthol, respectively (Figure 3B). Therefore, 10% was selected as the highest menthol concentration for subsequent experiments.

**Figure 3:**
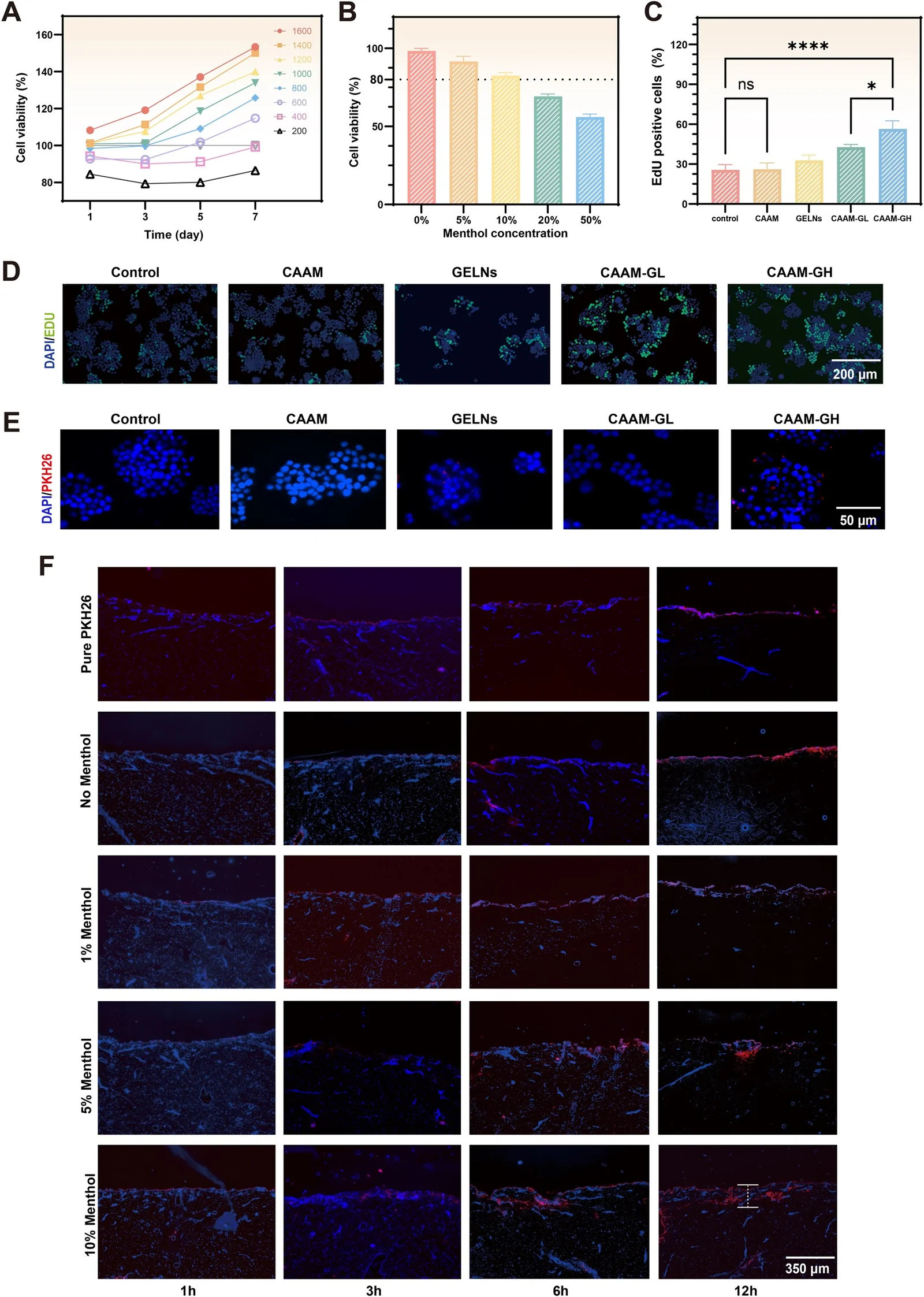
*In vitro* biocompatibility, proliferation, cellular uptake, and transdermal penetration. (**A**) Viability of HaCaT cells co-incubated with GELNs (0–1600 μg/mL) for 1, 3, 5 and 7 days. (**B**) Viability of HaCaT cells co-incubated with menthol-loaded CAAM hydrogels (0–50% menthol) for 24 h. (**C**) Quantitative analysis of EdU-positive cell proportions across treatment groups. (**D**) Representative fluorescence images of EdU-stained HaCaT cells. Scale bar: 200 μm. (**E**) Representative fluorescence images showing internalization of PKH26-labeled GELNs (red) by HaCaT cells after 6 h incubation; nuclei counterstained with DAPI (blue). Scale bar: 50 μm. (**F**) Representative fluorescence images of porcine skin cross-sections showing PKH26-labeled GELNs distribution at 1, 3, 6 and 12 h under pure PKH26, 0%, 1%, 5% and 10% menthol CAAM-GH. Scale bar: 350 μm. Data are presented as mean ± SD; ns, not significant; *p < 0.05, **p < 0.01, ***p < 0.001, ****p < 0.0001.

The EdU assay confirmed that CAAM-GH significantly promoted HaCaT cell proliferation compared with the control group (*P *< 0.0001), with significantly superior proliferative activity compared with CAAM-GL (*P *< 0.05). In contrast, the CAAM scaffold alone showed no significant effect (Figure 3C and D), indicating that the pro-proliferative effect was primarily driven by sustained GELNs release.

### Cellular uptake of GELNs

After 6 h of incubation, distinct PKH26 red fluorescence was observed in the perinuclear cytoplasmic region of HaCaT cells treated with GELNs, CAAM-GL and CAAM-GH groups, whereas no signal was detected in the control and CAAM groups (Figure 3E). The fluorescence intensity was visibly higher in the CAAM-GL and CAAM-GH groups than in the free GELNs group, suggesting that hydrogel-mediated release may prolong the extracellular availability of GELNs and thereby facilitate their sustained cellular uptake.

### *In vitro* transdermal penetration

As shown in Figure 3F, weak and sporadic red fluorescence was observed in the pure PKH26 group. Compared with the obvious time-dependent deep penetration of PKH26-labeled GELNs in the CAAM-PKH26-GELNs groups, the fluorescence signal of free PKH26 was mainly restricted to the superficial epidermal layer of porcine skin. This finding verified that the penetrating fluorescence detected in the hydrogel groups originated from PKH26-labeled GELNs rather than freely diffused PKH26 dye, confirming the reliability of the transdermal penetration results of GELNs. In the absence of menthol, only faint fluorescence was detected within the superficial skin layer throughout the 12-h observation period. The penetration depth increased with increasing menthol concentration. In the 1% menthol CAAM-GH group, the fluorescence signal extended into the superficial dermis after 6–12 h, whereas in the 5% menthol CAAM-GH group, it reached the mid-dermis as early as 3 h. The 10% menthol CAAM-GH group exhibited intense fluorescence within 1 h and reached a maximum penetration depth of ∼143 μm at 12 h, which was more than twice the measured epidermal thickness of the porcine skin model (62.7 ± 2.3 μm) and greater than that observed in the other groups (Figure 3F and Supplementary Figure S1) [30].

### Cell migration

The scratch assay demonstrated that the wound closure rates in the CAAM-GH group were significantly higher than those in all other groups at both 6 and 12 h (*P *< 0.05), with the scratch nearly completely closed by 12 h (Figure 4A). The CAAM and GELNs groups showed only limited wound closure, whereas CAAM-GL exhibited an intermediate wound closure rate. These findings suggest a concentration-dependent enhancement of cell migration associated with hydrogel-mediated GELNs delivery.

**Figure 4:**
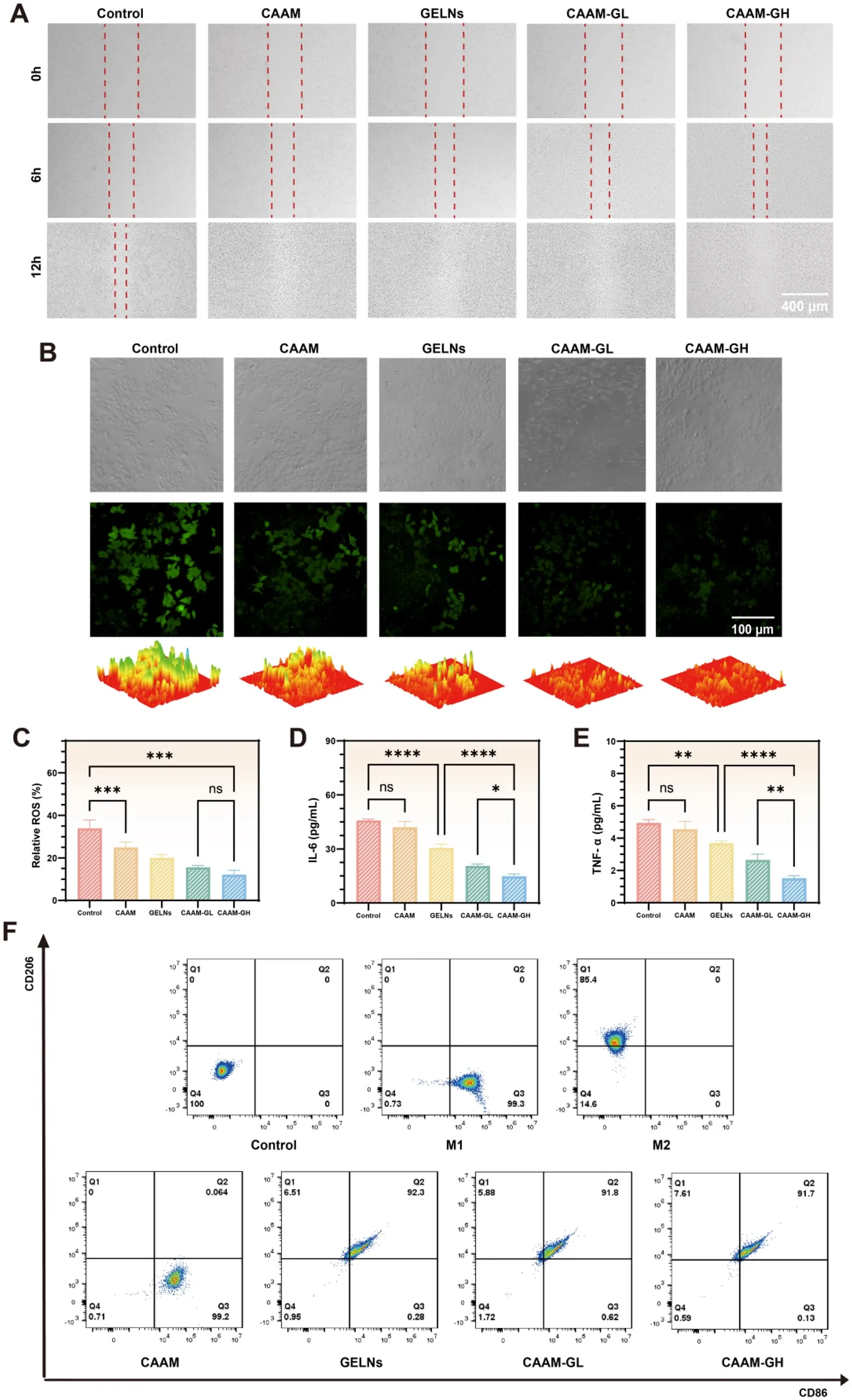
*In vitro* cell migration, ROS scavenging, anti-inflammatory effects, and macrophage polarization. (**A**) Representative scratch assay images of HaCaT cells at 0, 6 and 12 h. Scale bar: 400 μm. (**B**) Representative fluorescence images and 3D surface plots of DCFH-DA-stained HaCaT cells showing intracellular ROS levels. Scale bar: 100 μm. (**C**) Quantitative analysis of relative intracellular ROS levels. (**D**) ELISA quantification of IL-6 and (**E**) TNF-α in supernatants of LPS-stimulated RAW 264.7 cells. (**F**) Flow cytometric analysis of CD86 (M1 marker) and CD206 (M2 marker) expression in RAW 264.7 cells. Data are presented as mean ± SD; ns, not significant; *p < 0.05, **p < 0.01, ***p < 0.001, ****p < 0.0001.

### Intracellular ROS scavenging

DCFH-DA staining showed an overall decrease in green fluorescence intensity across the control, CAAM, GELNs, CAAM-GL and CAAM-GH groups (Figure 4B). Quantitative analysis confirmed ROS-associated fluorescence intensity was significantly reduced in all GELNs-containing groups compared with the control group (*P *< 0.001). The GELNs group also exhibited significantly lower ROS fluorescence intensity than the CAAM group (*P *< 0.001), whereas no significant difference was observed between the CAAM-GL and CAAM-GH groups (Figure 4C).

### Anti-inflammatory effects and macrophage polarization

ELISA analysis showed that the secretion of IL-6 and TNF-α by LPS-stimulated RAW 264.7 cells was significantly suppressed in the GELNs-containing groups, with CAAM-GH producing the greatest reduction compared with the control group (*P *< 0.0001 for both cytokines; Figure 4D and E). Flow cytometric analysis showed an overall increase in M2 macrophage polarization following treatment. CAAM-GH produced the highest proportion of CD206⁺/CD86⁻ M2 macrophages among all treatment formulations (7.61%), representing a significant increase compared with CAAM (0.11%) and the free GELNs group (6.51%), though remaining below the M2 positive control level (Figure 4F). The relatively modest degree of M2 polarization may reflect the difficulty of reversing LPS-induced M1 polarization and promoting M2 repolarization *in vitro*. Nevertheless, this finding is consistent with the more pronounced shift toward the M2 phenotype observed *in vivo* (section Immunohistochemical and immunofluorescence analysis).

### *In vivo* therapeutic efficacy

In the DNFB-induced AD mouse model, the CAAM-GH group showed progressive improvement from Day 7 onward, with marked alleviation of erythema, scaling and crusting by Day 15, resulting in a skin appearance similar to that of the normal group (Figure 5B). Dermatitis scores in the CAAM-GH group decreased continuously to ∼2 points by Day 17, significantly lower than those in all other treatment groups, including the dexamethasone reference group from Day 11 onward (Figure 5C). These findings indicate potent anti-inflammatory efficacy in this preclinical model. In contrast, the GELNs and CAAM-GL groups showed only limited improvement, with scores approaching 7 and 6 points, respectively, by Day 17. The greater improvement observed with CAAM-GH than with CAAM-GL suggests a GELNs loading-dependent therapeutic effect within the hydrogel delivery system.

**Figure 5:**
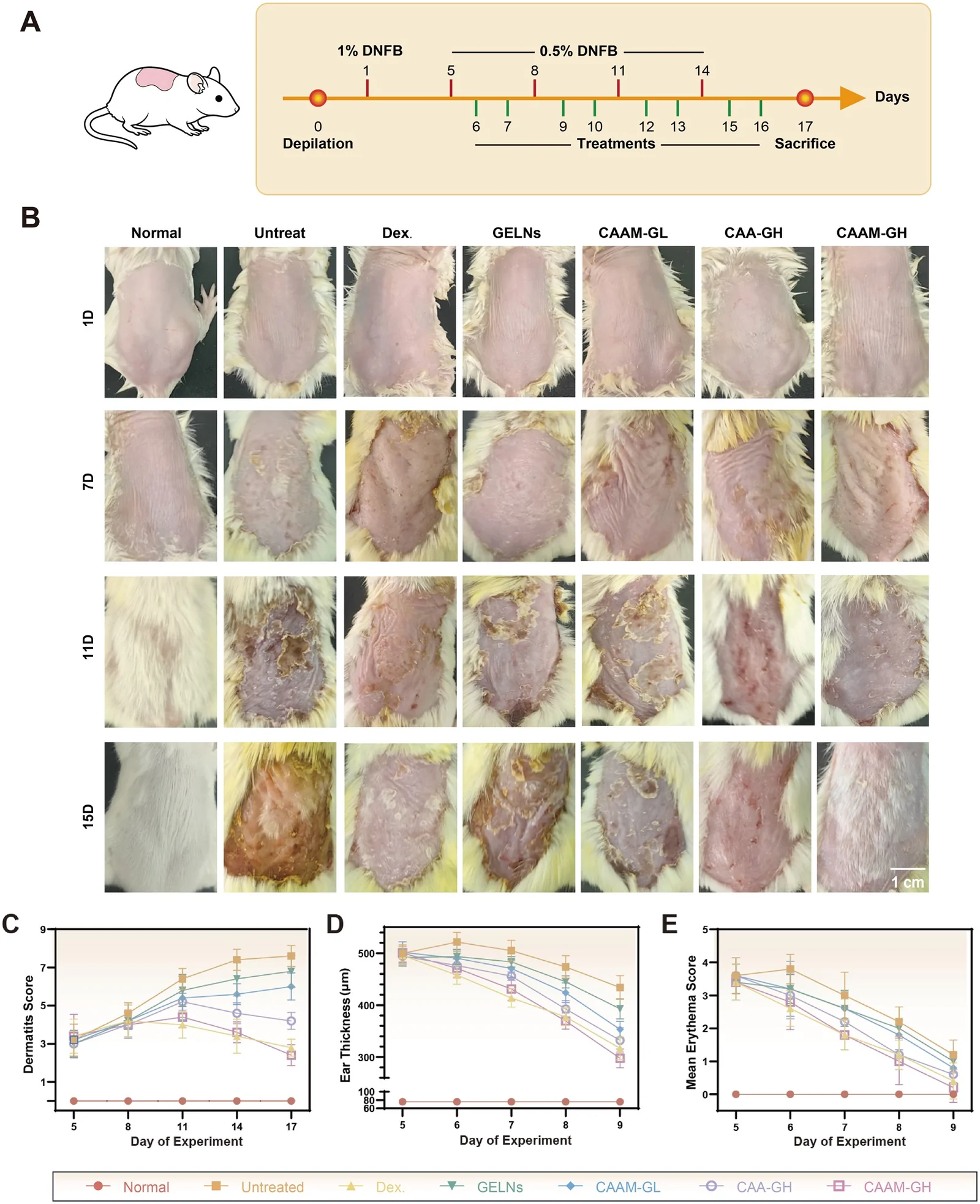
*In vivo* therapeutic efficacy in the DNFB-induced AD mouse model. (**A**) Schematic timeline of model induction and treatment protocol. (**B**) Representative photographs of dorsal skin lesions at Days 1, 7, 11 and 15. Scale bar: 1 cm. (**C**) Dermatitis scores over the experimental period. (**D**) Ear thickness and (**E**) mean erythema scores over the ear edema experiment.

### Ear edema model

From Day 6 onward, ear thickness and erythema scores in the CAAM-GH group were significantly lower than those in all other treatment groups and approached normal-group levels (Figure 5D and E and Supplementary Figure S2). CAAM-GH also produced greater reductions than dexamethasone, indicating potent suppression of local inflammatory manifestations.

### Histological analysis

As shown in the H&E staining results, the normal group exhibited an intact epidermal architecture with uniform thickness (Figure 6A and Supplementary Figure S3). In contrast, the untreated group displayed significant epidermal thickening and structural disorganization, which are typical AD-related pathological changes. The Dex. group showed a reduction in epidermal thickness, but epidermal structural abnormalities persisted. Among the GELNs-related treatment groups, the GELNs and CAAM-GL groups showed limited improvement in epidermal structure; however, the epidermal structure of the CAAM-GH group closely resembled that of the normal group, with thickness restored to a uniform state. Quantitative analysis showed that the epidermal thickness of the untreated group was significantly higher than that of the normal group (*P *< 0.0001), confirming the DNFB-induced epidermal hyperplastic effect. Notably, the epidermal thickness in the CAAM-GH group did not differ significantly from that in the normal group and was significantly lower than that in the CAAM-GL group (*P *< 0.001), indicating its efficacy in reversing AD-associated epidermal hyperplasia.

**Figure 6:**
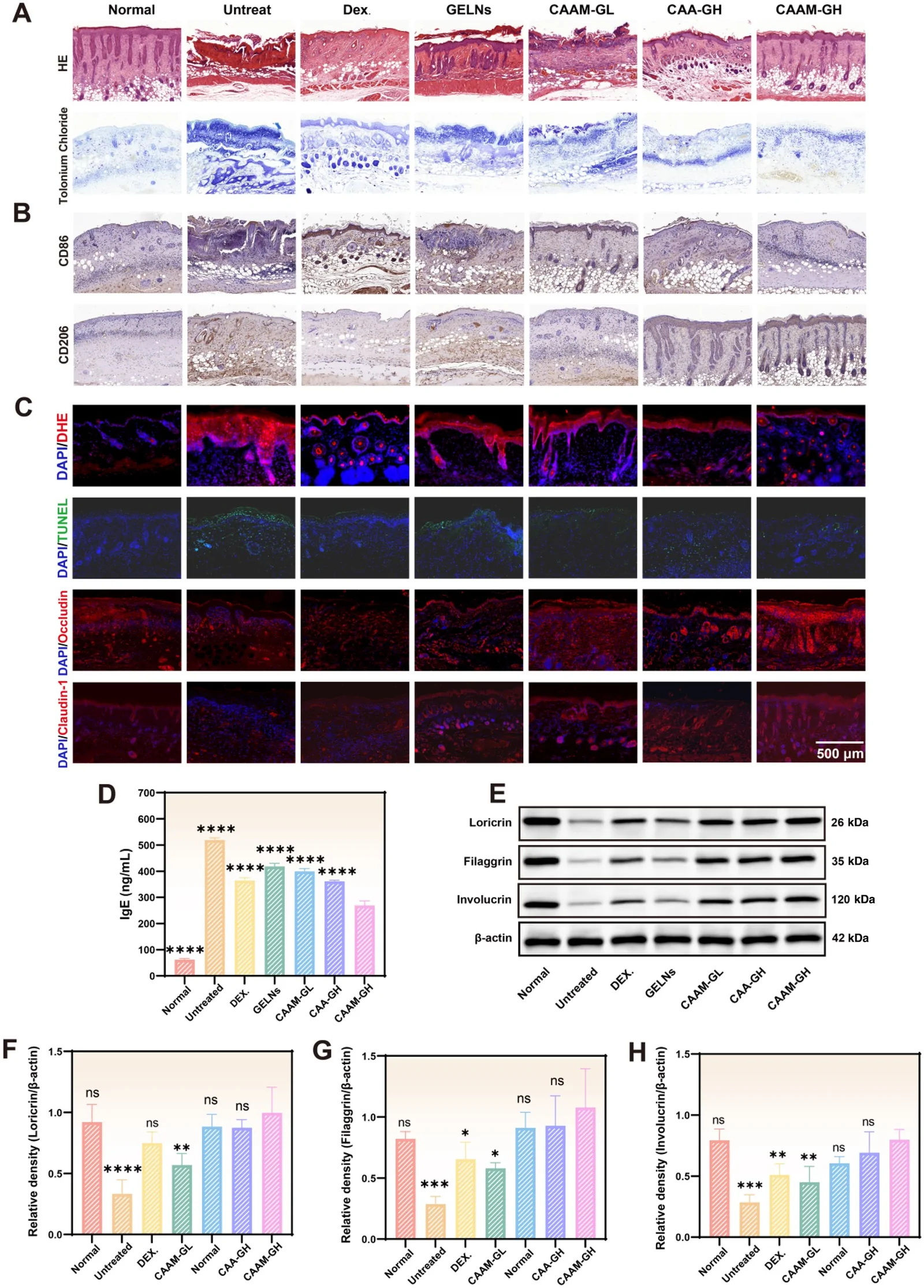
Histopathological and immunofluorescence analysis of AD skin tissue. (**A**) Representative H&E staining (upper) and Toluidine Blue staining (lower) of dorsal skin sections. Scale bar: 500 μm. (**B**) Representative IHC staining for CD86 (M1 marker, upper) and CD206 (M2 marker, lower). Scale bar: 500 μm. (**C**) Representative IF staining for DHE (ROS, row 1), TUNEL (apoptosis, row 2), occludin-1 (row 3), and claudin-1 (row 4); nuclei counterstained with DAPI (blue). Scale bar: 500 μm. (**D**) ELISA for total serum IgE. (**E**) Representative Western blot images of loricrin, filaggrin and involucrin. Quantitative relative density analysis of (**F**) Loricrin, (**G**) Filaggrin, (**H**) Involucrin normalized to β-actin. Data are presented as mean ± SD; ns, not significant; *p < 0.05, **p < 0.01, ***p < 0.001, ****p < 0.0001.

### Immunohistochemical and immunofluorescence analysis

IHC staining showed markedly reduced CD86 expression and substantially increased CD206 expression in skin treated with CAAM-GH, resulting in the highest CD206/CD86 ratio among all groups (Figure 6B and Supplementary Figure S4). In the untreated group, CD86 staining was markedly increased, whereas the CD206 staining remained weak, indicating predominantly pro-inflammatory, M1-like macrophage phenotype. The Dex. group showed a slight reduction in CD86 expression but limited improvement in CD206 expression. In contrast, the CAAM-GH group exhibited lower CD86 expression and higher CD206 expression than the GELNs, CAAM-GL and CAA-GH groups.

DHE staining revealed intense red fluorescence in the untreated group, indicating elevated ROS-associated oxidative stress in AD skin tissue (Figure 6C and Supplementary Figure S5). The DHE fluorescence signal was significantly attenuated in the CAAM-GH group, reaching levels comparable to the normal group. TUNEL staining showed abundant TUNEL-positive signals in the untreated group, whereas the CAAM-GH group exhibited a significant reduction in TUNEL positivity, with only scattered apoptotic cell signals observed (Supplementary Figure S6). The attenuation of ROS signals and reduction in apoptotic cells in the CAAM-GH group suggest that CAAM-GH alleviates oxidative stress and associated apoptosis in AD skin tissue.

IF staining for occludin-1 and claudin-1 revealed significantly enhanced and more continuous fluorescence signals in the CAAM-GH group, closely resembling those observed in the normal group (Figure 6C and Supplementary Figures S7 and S8). The Dex. group showed partial recovery of occludin-1 and claudin-1 signals, but the signals remained discontinuous. Occludin and claudin-1 are key tight junction proteins involved in maintaining skin barrier integrity. Their sparse and disorganized expression in the untreated group was consistent with substantial barrier disruption, whereas their enhanced and more continuous distribution in the CAAM-GH group supported the potential of CAAM-GH to promote skin barrier restoration.

### Analysis of serum IgE levels

Serum IgE was detected by ELISA to assess the inflammation of the DNFB-induced AD mice (Figure 6D). Serum IgE concentrations were substantially higher in the untreated AD group relative to the normal group, verifying the successful construction of the DNFB-induced AD mouse model. Compared with other groups, CAAM-GH significantly reduced serum IgE (*P *< 0.0001). The CAAM-GH group achieved the lowest IgE level across all hydrogel-based formulations.

### Western blot analysis of key epidermal barrier-related proteins

Western blot was performed to detect the expression of key epidermal barrier proteins including loricrin, filaggrin and involucrin (Figure 6E–H). Compared with the CAAM-GH treatment group, the untreated group exhibited significantly downregulated protein levels of loricrin, filaggrin and involucrin, confirming severe disruption of epidermal barrier function in DNFB-induced lesions. Other treatment groups, such as Dex., only achieved limited upregulation of these barrier proteins. By contrast, CAAM-GH treatment effectively recovered the abundance of filaggrin, loricrin and involucrin to levels comparable to those in normal mice.

### Biosafety assessment

To further evaluate the biosafety of CAAM-G, hemolysis tests were conducted. The results indicated that Dex, GELNs, CAAM-GL, CAA-GH and CAAM-GH did not increase the hemolysis rate (Figure 7A and B). There were no significant differences in H&E staining results of the heart, lung, kidney, spleen and liver among different groups (Figure 7C). Serum biochemical detection showed that the levels of ALT, AST, BUN and CRE in the CAAM-GH group were markedly lower than those in the untreated model group and Dex group, revealing that CAAM-GH does not induce extrahepatic and renal damage (Supplementary Figures S9–S12). These results suggest that CAAM-G is biologically safe.

**Figure 7:**
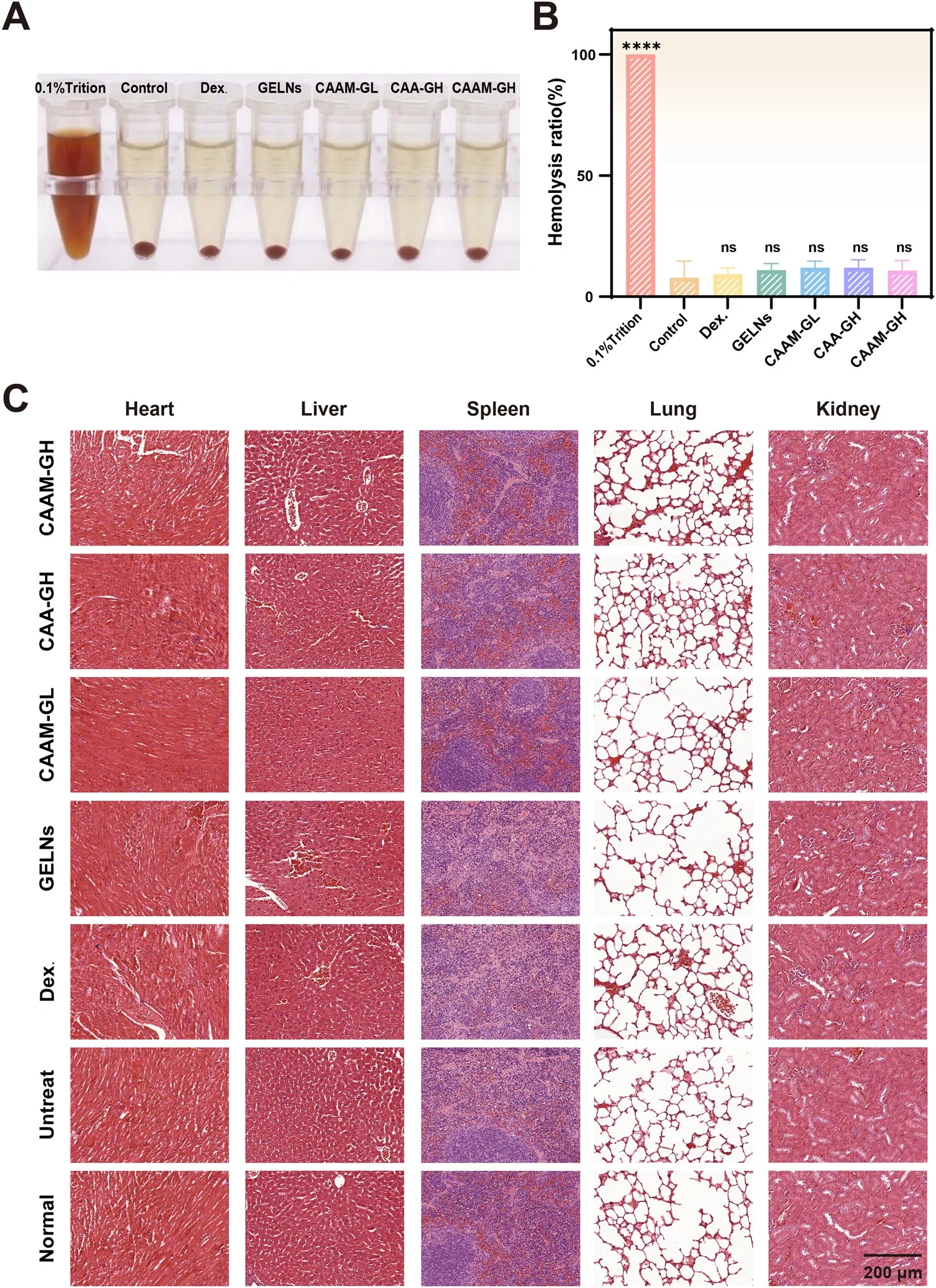
Safety evaluation. (**A**) Photographs of hemolysis test; 0.1% Triton X-100 as a positive control. (**B**) Hemolysis ratio of erythrocytes incubated with test formulations. (**C**) Representative H&E staining of major organs (heart, liver, spleen, lung, kidney) from each treatment group. Scale bar: 200 μm.

### Transcriptomic analysis and Western blot analysis of key signaling pathways

Whole-transcriptome sequencing identified 2529 DEGs between the CAAM-GH and untreated groups, including 1243 upregulated and 1286 downregulated genes (|log_2_ fold change| > 1, *P *< 0.05; Figure 8A and B). Correlation heatmap analysis showed high within-group consistency (Figure 8C). GO enrichment analysis revealed significant enrichment of terms related to ‘canonical NF-κB signal transduction’, ‘IκB phosphorylation’, ‘JAK–STAT receptor signaling’ and ‘regulation of tumor necrosis factor superfamily cytokine production’ (Figure 8D). KEGG analysis identified the NF-κB signaling pathway as the pathway with the highest enrichment ratio (rich factor > 0.24), followed by the TNF, JAK–STAT and PI3K–Akt signaling pathways (Figure 8E). GSEA revealed significant negative enrichment of the NF-κB, TNF and JAK–STAT inflammatory signaling pathways, together with positive enrichment of the AMPK and cAMP signaling pathways (Figure 8F). These findings suggest enhancement of pathways associated with protective metabolic and homeostatic mechanisms; however, additional protein-level validation is required to confirm most of these pathway-level changes.

**Figure 8:**
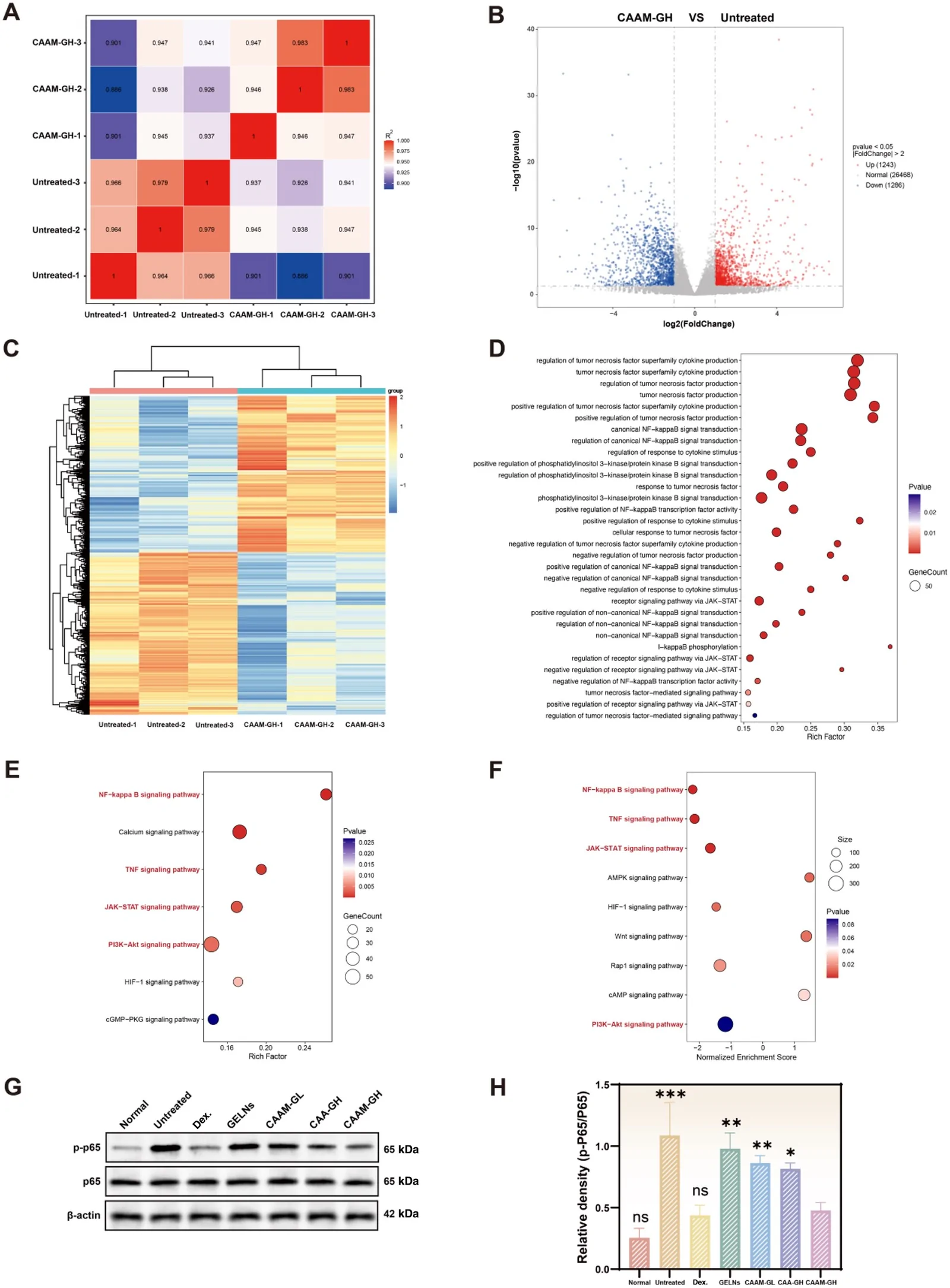
Transcriptomic and molecular mechanism analysis. (**A**) Sample correlation heatmap of untreated and CAAM-GH groups. (**B**) Volcano plot of DEGs (|log_2_FoldChange| > 1, *P *< 0.05). (**C**) Hierarchical clustering heatmap of DEGs. (**D**) GO functional enrichment analysis (biological process). (**E**) KEGG pathway enrichment analysis. (**F**) GSEA enrichment scores for selected signaling pathways. (**G**) Representative Western blot images of p-p65, p65 and β-actin. (**H**) Quantitative analysis of p-p65/p65 ratio.

Western blotting showed that the p-p65/p65 ratio in CAAM-GH-treated skin did not differ significantly from that in the normal group and was significantly lower than those in all other treatment groups, including the dexamethasone reference group (Figure 8G and H). These results provide protein-level evidence that CAAM-GH inhibits NF-κB pathway activation, which may contribute to its therapeutic effects.

## Discussion

To overcome the above bottlenecks limiting the clinical translation of plant-derived nanovesicle preparations for AD management, the present study proposes three innovative design strategies. First, *G.macrophylla* Pall.-derived GELNs are investigated for the first time as novel PENs for anti-AD applications, expanding the resource bank of plant-derived extracellular vesicle-like nanomedicines beyond the commonly studied edible herbs. Second, L-menthol is covalently anchored into the hydrogel network via chemical bonding instead of simple physical mixing, enabling stepwise pH-triggered release of menthol and GELNs in response to the alkaline microenvironment of inflammatory AD lesions. Third, the integrated hydrogel formulation combines transdermal penetration promotion, *in situ* sustained drug release, antioxidant and immunoregulatory functions into a single corticosteroid-free topical scaffold, synchronously targeting oxidative stress, abnormal macrophage polarization and impaired epidermal barrier function in the AD pathological cascade. Such integrated structural and functional design avoids repeated multi-agent medication and systemic adverse reactions caused by long-term corticosteroid administration, providing a feasible innovative paradigm for developing PENs loaded stimuli-responsive wound dressings against inflammatory dermatosis.

To validate the first design rationale, namely the use of *G.macrophylla*-derived nanovesicles as bioactive botanical carriers, we first characterized the physicochemical properties of isolated GELNs. The physicochemical profile of the isolated GELNs—uniform vesicular morphology, a narrow size distribution centered at 132.62 nm, and a strongly negative zeta potential of −32.46 mV—is consistent with established characterization criteria for plant-derived extracellular vesicles as recommended by current consensus guidelines (Figure 1A–D) [10, 31]. A zeta potential more negative than −30 mV is generally considered to indicate sufficient electrostatic repulsion to ensure colloidal stability during storage and formulation, a prerequisite for reproducible biological activity [32]. It is noteworthy that plant-derived nanovesicles generally lack classical mammalian exosome marker proteins, such as CD63 and TSG101; therefore, their characterization primarily relies on morphological and physicochemical parameters, including vesicular morphology, particle size distribution and surface charge, as applied in this study [10, 33]. Proteomic and metabolomic characterization of GELNs cargo, which would provide deeper molecular identity and enable the identification of specific bioactive constituents, represents a priority for future work [34].

After confirming the vesicular identity and colloidal stability of GELNs, we next examined whether these nanovesicles possessed intrinsic biological activity relevant to AD pathology. The concentration-dependent radical scavenging activity observed across four assay systems establishes GELNs as intrinsically bioactive particles rather than passive carriers, confirming dose-dependent antioxidant capacity against multiple ROS, which is relevant to the oxidative stress environment of AD lesional skin (Figure 1F–I). This is mechanistically consistent with the known pharmacology of *G.macrophylla* bioactive constituents, particularly gentiopicroside, which suppresses ROS generation via the ROS–NF-κB–NLRP3 axis [14]. In the context of AD, where excessive ROS accumulation perpetuates keratinocyte damage and amplifies M1 macrophage polarization [6, 35, 36], this intrinsic antioxidant capacity positions GELNs as therapeutically active components of the delivery system rather than inert carriers.

Although GELNs exhibited intrinsic antioxidant activity, their therapeutic application in AD lesions requires an appropriate topical matrix capable of improving retention, controlled release and skin penetration. Therefore, we constructed a menthol-integrated CAAM hydrogel system. The appearance of the menthol-associated C–O stretching band at 1160 cm^−1^ in FTIR analysis supports the covalent integration of menthol into the polymer network rather than simple physical entrapment, which is critical for ensuring controlled release kinetics rather than burst release (Figure 2A) [29]. However, because FTIR alone may not be sufficient to unequivocally prove covalent bonding, this conclusion should be interpreted together with the synthetic design and other structural evidence. The pore size is inversely correlated with cross-linking density in polyacrylamide-based systems. This property explains the tunable porous architecture, which facilitates uniform GELNs distribution, exudate absorption and regulated cargo release—properties that are collectively important for skin dressing applications [19, 20].

Beyond chemical integration and porous architecture, the mechanical and swelling properties of the hydrogel are equally critical for its performance as a topical dressing. The selection of CAAM233 reflects an optimal balance among mechanical strength (peak tensile stress >55 kPa), extensibility and an equilibrium SR >900% (Figure 2D–I). These properties enable synchronous deformation with physiological skin movement without debonding, directly supporting wearer comfort and long-term adhesive durability [19]. The high swelling capacity further enables efficient exudate absorption and helps maintain a moist wound microenvironment conducive to epithelial repair [37, 38].

In addition to mechanical adaptability and fluid management, lesion-responsive release is essential for achieving site-preferential therapy in AD. The pH-dependent release behavior is mechanistically governed by the ionization equilibrium of pendant carboxyl groups: protonated –COOH groups at pH 5.5 promote intermolecular hydrogen bonding that tightens the network, while progressive deprotonation at pH 8.5 generates electrostatic repulsion that drives network expansion and widens diffusion pathways [20, 39]. The selection of pH 8.5 as an alkaline lesional test condition is supported by multiple clinical measurements reporting elevated skin surface pH in AD lesions, with values commonly reported in the range of 6.0–7.5 in clinical measurements, and further increased toward pH 7.5–8.5 at sites of severe inflammation [39, 40]. Thus, pH 8.5 should be interpreted as a stringent upper-bound condition for evaluating pH-responsive release rather than a universal pH value for all AD lesions. The intermediate release profiles observed at pH 6.0, 6.5, 7.0, 7.5 and 8.0 (Figure 2H–I) further indicate that clinically relevant drug liberation would occur across a broad spectrum of lesional pH values, enabling a graded therapeutic response proportional to inflammatory severity. This pH-gating mechanism has been validated in several carboxyl-functionalized hydrogel systems for inflammatory skin disease models [20], and its implementation here enables lesion-preferential drug release while minimizing premature release at normal skin pH, thereby reducing potential off-lesion exposure.

However, controlled release alone is insufficient for topical nanotherapy, because the stratum corneum remains the major barrier to effective delivery into lesional skin. The stratum corneum represents the primary physiological barrier limiting transdermal delivery of nanoscale particles [21, 41]. The concentration-dependent enhancement of GELNs penetration by menthol is consistent with its well-characterized mechanism of disrupting ordered SC lipid packing by increasing bilayer fluidity [21, 42]. The 10% menthol formulation achieved a maximum penetration depth of 143 μm within 12 h in the porcine skin model—effectively traversing the full epidermis (62.7 μm) and reaching the superficial dermis where dermal fibroblasts and resident immune cells reside (Figure 3F, Supplementary Figure S1) [30]. This concentration represents a deliberate optimum: lower concentrations (1%, 5%) provided insufficient penetration enhancement, while higher concentrations (20%, 50%) compromised cell viability below the 80% threshold (Figure 3B). It should be noted that menthol at higher concentrations may also exert direct anti-inflammatory effects independent of its penetration-enhancing function [43]. However, whether 10% menthol itself contributes additively or synergistically to the anti-inflammatory effect of CAAM-G requires further investigation. The identification of 10% as the optimal concentration nonetheless provides a precise and translatable formulation parameter.

Having demonstrated enhanced skin penetration, we further evaluated whether GELNs released from the hydrogel could be efficiently internalized by keratinocytes and promote barrier-repair-related cellular behaviors. Perinuclear accumulation of PKH26-labeled GELNs in HaCaT cells supports efficient endocytic internalization, consistent with uptake mechanisms reported for other plant-derived nanovesicles in epithelial models [10]. The greater fluorescence intensity in the CAAM-GH group than in the free GELNs group suggests the sustained-release advantage of the hydrogel carrier, which maintains a continuous extracellular concentration gradient favoring ongoing cellular uptake. This advantage also helps explain the excellent pro-proliferative and pro-migratory effects observed in EdU and scratch assays. Enhanced keratinocyte proliferation and migration are mechanistically relevant to AD therapy, as impaired keratinocyte motility contributes to delayed barrier repair in chronic AD lesions [3].

Because AD progression is driven not only by impaired keratinocyte repair but also by dysregulated immune activation, we next assessed the immunomodulatory effects of GELNs-containing formulations in macrophages. The significant suppression of IL-6 and TNF-α secretion and promotion of M2 macrophage polarization in GELNs-containing formulations confirm that the anti-inflammatory function of CAAM-G is primarily driven by GELNs bioactive components rather than the CAAM scaffold alone (Figure 4D and E) [44–46]. The excellent performance of CAAM-GH over free GELNs at equivalent nominal concentrations reflects the sustained-release advantage of the hydrogel carrier, which maintains effective GELNs concentrations at the cell surface throughout the 24-h treatment period—an effect that free GELNs cannot sustain, as they may undergo rapid dilution, clearance, or instability under culture conditions [47, 48]. It should be noted that the absolute proportion of CD206⁺/CD86⁻ M2 macrophages achieved by CAAM-GH *in vitro* (7.61%) remained substantially below the M2 positive control. This is consistent with the inherent difficulty of fully reversing polarization in strongly LPS-stimulated macrophage models (Figure 4F); the progressive shift in M2/M1 ratio nonetheless represents a biologically meaningful improvement, and the more pronounced polarization shift observed *in* vivo (section Immunohistochemical and immunofluorescence analysis) suggests that the full therapeutic effect of CAAM-G is better captured in the physiologically relevant lesional microenvironment [49, 50].

The macrophage-polarizing effect of CAAM-GH may be closely linked to its antioxidant activity, as oxidative stress is an upstream driver of inflammatory macrophage activation. The broad *in vitro* antioxidant performance of GELNs provides a vital mechanistic underpinning for the *in vivo* anti-inflammatory effects of CAAM-GH. The multi-system verified ROS-scavenging capability of GELNs enables local suppression of oxidative stress within lesional tissue after transdermal delivery. The potent ROS-scavenging activity of CAAM-GH represents not only a direct antioxidant effect but also a mechanistically upstream intervention: by interrupting the ROS–NF-κB–M1 polarization amplification loop, ROS attenuation may independently contribute to the macrophage polarization shift observed downstream [51, 52]. This dual action—direct cytokine suppression and upstream ROS-mediated polarization modulation—distinguishes CAAM-G from formulations targeting either oxidative stress or inflammation alone. Compared with *Portulaca oleracea*-derived nanovesicles, which modulate M1/M2 balance via NF-κB and STING pathway inhibition but require microneedle-facilitated delivery to overcome the transdermal barrier [13], CAAM-G achieves comparable immunological endpoints through an entirely non-invasive pH-responsive hydrogel platform, offering a practical advantage for chronic disease management where repeated application is required.

The *in vitro* antioxidant, pro-repair and immunomodulatory effects of CAAM-GH were further translated into pronounced therapeutic efficacy in the DNFB-induced AD mouse model. The excellent performance of CAAM-GH compared with the dexamethasone reference in the DNFB-induced AD mouse model—with dermatitis scores reduced to ∼2 points by Day 17 and ear edema approaching normal levels from Day 6—provides strong preclinical evidence for the therapeutic potential of this platform (Figure 5B–E). The DNFB model reliably recapitulates the immune dysregulation, epidermal barrier dysfunction and inflammatory cell infiltration and is widely used for preclinical evaluation of anti-AD interventions [26, 27]. The direct comparison between CAAM-GH and CAA-GH isolates the functional contribution of menthol-mediated transdermal enhancement *in vivo*, while the comparison with CAAM-GL confirms a dose-dependent GELNs therapeutic effect. While well established, the DNFB model is fundamentally a hapten-induced, contact hypersensitivity-based AD-like dermatitis model and therefore does not fully recapitulate the chronic, relapsing nature and complex immunopathology of human AD; validation in additional preclinical models and ultimately in clinical studies will be necessary to confirm translational efficacy.

Consistent with the macroscopic improvement, histological and molecular analyses further confirmed restoration of the lesional skin microenvironment. Histological normalization of epidermal thickness and mast cell infiltration, combined with *in vivo* M2 polarization shift, ROS attenuation, reduced apoptosis, restoration of occludin-1 and claudin-1 tight junction protein expression and elevated levels of filaggrin, loricrin and involucrin confirmed via Western blot, collectively provide multi-level tissue evidence for functional skin barrier repair [53–55]. The restoration of tight junction proteins and cornified envelope proteins is particularly significant, as their downregulation in AD is a well-documented consequence of inflammation and oxidative stress [2, 56, 57], and their recovery indicates that the anti-inflammatory and antioxidant effects of CAAM-G translate into meaningful structural reconstitution of the epidermal barrier rather than merely suppressing surface symptoms.

To further elucidate the molecular basis underlying these phenotypic improvements, transcriptomic enrichment analyses were performed. The convergence of GO, KEGG and GSEA analyses on NF-κB as the central regulatory pathway is mechanistically coherent: NF-κB is the central transcriptional hub driving inflammatory mediator production in AD, and GO enrichment of ‘IκB phosphorylation’ suggests that CAAM-GH may interfere with this regulatory step [3, 58]. This is consistent with the known mechanism of gentiopicroside, a major bioactive constituent of *G.macrophylla*, which inhibits IKKβ phosphorylation to block NF-κB nuclear translocation and downstream pro-inflammatory gene expression [14]. Western blot confirmation that p-p65/p65 ratios in CAAM-GH-treated skin were restored to normal group levels provides direct protein-level validation of NF-κB pathway suppression as a central mechanism of action (Figure 8G and H). The concurrent negative enrichment of JAK-STAT and TNF pathways further indicates multi-pathway targeting consistent with the multi-component bioactive nature of GELNs. The positive enrichment of AMPK and cAMP pathways at the transcriptomic level suggests potential activation of protective metabolic and mast cell-stabilizing mechanisms; however, as these pathway-level findings have not yet been confirmed at the protein level, they should be interpreted as hypothesis-generating observations warranting dedicated validation in future studies. In addition, transcriptomic analyses revealed robust enrichment of AMPK and cAMP pathways, which contribute to the restored expression of filaggrin, loricrin and involucrin in AD skin by dual mechanisms. Activated AMPK directly stabilizes occludin/claudin complexes and inhibits NF-κB-driven inflammatory cytokine production that degrades skin barrier structural proteins. Elevated cAMP further reinforces keratinocyte intercellular adhesion to reverse inflammation-induced junction disruption. Combined suppression of the NF-κB cascade by AMPK creates a synergistic barrier-protective effect, linking transcriptomic pathway changes to the *in vivo* epidermal structural repair observed in our DNFB AD model, consistent with published signaling data in AD [59, 60].

Despite these encouraging findings and mechanistic insights, several limitations of this study should be acknowledged. First, the physicochemical characterization of GELNs, while consistent with current standards for plant-derived extracellular vesicles, does not include proteomic or metabolomic profiling; the specific bioactive components responsible for the observed antioxidant, anti-inflammatory and macrophage-polarizing effects remain to be identified, and such characterization will be essential for establishing structure-activity relationships and ensuring batch-to-batch reproducibility. Second, the *in vitro* M2 polarization data should be interpreted in light of the strongly polarized LPS-stimulated RAW 264.7 model, which may not fully recapitulate the dynamic immune microenvironment of AD lesions; the modest absolute M2 proportion achieved *in vitro* (7.61%) is better considered alongside the more pronounced polarization shift observed in histological analysis of treated skin. Third, the present safety evaluation provides initial biocompatibility evidence but does not constitute systematic toxicological assessment; comprehensive studies including repeated-dose toxicology and skin sensitization testing will be required prior to clinical translation. Finally, while the DNFB model reliably recapitulates key features of human AD, validation in additional preclinical models and clinical studies will be necessary to confirm translational efficacy.

## Conclusion

This study develops CAAM-G, an innovative pH-responsive multifunctional hydrogel platform that integrates GELNs and L-menthol into a carboxymethyl cellulose-based matrix for corticosteroid-free topical therapy of AD. The major innovation lies in the combined use of previously unexplored *G.macrophylla*-derived botanical nanovesicles as bioactive therapeutic components, covalently incorporated menthol as both a penetration enhancer and release-regulating module, and a lesion-responsive hydrogel scaffold capable of sustained drug release in the alkaline inflammatory microenvironment of AD lesions. Through this integrated design, CAAM-G simultaneously overcomes the key translational barriers of plant-derived nanovesicle therapy, including poor skin penetration, insufficient retention and uncontrolled release, while exerting coordinated antioxidant, anti-inflammatory, macrophage-polarizing and barrier-repair effects mainly through ROS–NF-κB-related pathway modulation. Therefore, this work not only proposes a promising corticosteroid-free therapeutic strategy for AD but also establishes a new design paradigm for stimuli-responsive, plant-derived nanovesicle-loaded hydrogel dressings for inflammatory skin diseases.

## Supplementary Material

rbag196_Supplementary_Data
