# Supplementary material for: pH-responsive hydrogel facilitates skin penetration of extracellular vesicle-like nanovesicles for enhanced atopic dermatitis therapy
Source: Regen Biomater. 2026 Sep 3;13:rbag196. doi: 10.1093/rb/rbag196 (PMC13730573; doi:10.1093/rb/rbag196)
Supplement: Supplementary file 1 [file rbag196_supplementary_data.docx]

Supporting Information

**Figure S1.** Time-dependent transdermal penetration depth of PKH26-labeled GELNs across porcine skin at pure PKH26, 0%, 1%, 5%, and 10% menthol CAAM-GH concentrations over 12 h.

**Figure S2.** Representative photographs of mouse ear before and after DNFB-induced ear edema.

**Figure S3.** Quantitative analysis of epidermal thickness across treatment groups.

**Figure S4.** Quantitative analysis of M2/M1 cell ratio in dorsal skin sections across treatment groups.

**Figure S5.** Quantitative analysis of relative ROS levels in dorsal skin sections assessed by DHE staining.

**Figure S6.** Quantitative analysis of relative TUNEL-positive cell proportions across treatment groups.

**Figure S7.** Quantitative analysis of relative occludin-1 expression across treatment groups.

**Figure S8.** Quantitative analysis of relative claudin-1 expression across treatment groups.

**Figure S9.** Serum ALT level.

**Figure S10.** Serum AST level.

**Figure S11.** Serum BUN level.

**Figure S12.** Serum CRE level.
